# Liraglutide protects cardiac function in diabetic rats through the PPARα pathway

**DOI:** 10.1042/BSR20180059

**Published:** 2018-03-16

**Authors:** Qian Zhang, Xinhua Xiao, Jia Zheng, Ming Li, Miao Yu, Fan Ping, Tong Wang, Xiaojing Wang

**Affiliations:** Key Laboratory of Endocrinology, Ministry of Health, Department of Endocrinology, Peking Union Medical College Hospital, Peking Union Medical College, Chinese Academy of Medical Sciences, Beijing 100730, China

**Keywords:** cardiac function, Diabetes, glucagon-like peptide-1 analogue, PPAR

## Abstract

Increasing evidence shows that diabetes causes cardiac dysfunction. We hypothesized that a glucagon-like peptide-1 (GLP-1) analog, liraglutide, would attenuate cardiac dysfunction in diabetic rats. A total of 24 Sprague–Dawley (SD) rats were divided into two groups fed either a normal diet (normal, *n*=6) or a high-fat diet (HFD, *n*=18) for 4 weeks. Then, the HFD rats were injected with streptozotocin (STZ) to create a diabetic rat model. Diabetic rats were divided into three subgroups receiving vehicle (diabetic, *n*=6), a low dose of liraglutide (Llirag, 0.2 mg/kg/day, *n*=6), or a high dose of liraglutide (Hlirag, 0.4 mg/kg/day, *n*=6). Metabolic parameters, systolic blood pressure (SBP), heart rate (HR), left ventricular (LV) function, and whole genome expression of the heart were determined. Diabetic rats developed insulin resistance, increased blood lipid levels and oxidative stress, and impaired LV function, serum adiponectin, nitric oxide (NO). Liraglutide improved insulin resistance, serum adiponectin, NO, HR, and LV function and reduced blood triglyceride (TG), total cholesterol (TC) levels, and oxidative stress. Moreover, liraglutide increased heart nuclear receptor subfamily 1, group H, member 3 (*Nr1h3*), peroxisome proliferator activated receptor (*Ppar*) *α (Pparα)*, and *Srebp* expression and reduced diacylglycerol O-acyltransferase 1 (*Dgat*) and angiopoietin-like 3 (*Angptl3*) expression. Liraglutide prevented cardiac dysfunction by activating the PPARα pathway to inhibit *Dgat* expression and oxidative stress in diabetic rats.

## Introduction

Diabetes is a worldwide public health problem that has prevalence greater than 5.71% in adults [1]. Chronic hyperglycemia leads to a high risk of cardiovascular events [2]. Cardiovascular disease is a leading cause of morbidity and mortality worldwide. Diabetic cardiomyopathy (DCM) is defined as cardiac hypertrophy that is independent of hypertension and coronary artery disease (CAD). The three main risk factors of DCM are insulin resistance, hyperinsulinemia, and hyperglycemia. To date, some cellular and molecular defects, including impaired insulin signaling, hyperglycemia, glucotoxicity, cardiac lipotoxity, mitochondrial dysfunction, oxidative stress, endoplasmic reticulum (ER) stress, and cardiomyocyte apoptosis, have been reported as primary causes of DCM pathogenesis [3,4]. Treatments providing glycemic control and cardiovascular protection are important to improve the health of people all over the world [5].

Glucagon-like peptide-1 (GLP-1) is secreted from L-cells in the gut. In addition, to controlling blood glucose levels, GLP-1 also reduces gastric emptying and inhibits appetite. However, GLP-1 can be digested quickly by dipeptidyl peptidase-4 (DPP-4). In clinical practice, the GLP-1 receptor (GLP-1R) agonist liraglutide was effective at controlling blood glucose levels. In addition to pancreatic α and β cells, GLP-1Rs are also found in the heart. More and more clinical trials and animal experiments have shown evidence of the protective cardiac effects of liraglutide, independent of its effects on blood glucose levels. Short-term liraglutide treatment mildly improves left ventricular ejection fraction (LVEF) in ST-segment elevation myocardial infarction patients [6]. In type 1 diabetic rats, liraglutide inhibits cardiac steatosis, oxidative stress, and apoptosis through activating the activated protein kinase (AMPK)-sirtuin 1 (Sirt1) pathway [7]. However, the exact mechanism of the beneficial effects of liraglutide on cardiac tissue in diabetic rats remains to be elucidated.

Therefore, the present study aimed to investigate whether liraglutide has protective cardiac effects and its underlying mechanism in type 2 diabetic rats. We employed a global microarray analysis combined with bioinformatics to explore key genes and pathways affected by liraglutide in cardiac tissue from diabetic rats.

## Materials and methods

### Animal treatments and diets

Five-week-old male Sprague–Dawley (SD) rats, provided by the Institute of Laboratory Animal Science, Chinese Academy of Medical Sciences and Peking Union Medical College (Beijing, China, SCXK-2014-0013), were maintained in a pathogen-free environment with a 12-h light/dark cycle and free access to food and water. Animal experiments followed the Guide for the Care and Use of Laboratory Animals published by the U.S. National Institutes of Health (NIH publication number 85-23, revised 1996) and were approved by the Animal Care Committee of the Peking Union Medical Hospital Animal Ethics Committee (Project XHDW-2015-0051, 15 February 2015). After acclimatization, the rats were randomly divided into four groups (*n*=6 for each group): control, diabetic, low-dose liraglutide (Llirag), and high-dose liraglutide (Hlirag) groups. The control group was fed a standard rodent diet (kcal%: 10% fat, 20% protein, and 70% carbohydrate; 3.85 kcal/g); the other three groups were fed a high-fat diet (HFD) (kcal%: 45% fat, 20% protein, and 35% carbohydrate; 4.73 kcal/g, Research Diet, New Brunswick, NJ, U.S.A.). After 4 weeks of HFD feeding, diabetes was induced in the rats by streptozotocin (STZ, 30 mg/kg) injection. Fasting blood glucose levels higher than 11.1 mmol/l were considered standard for the diabetic model. Then, 0.2 or 0.4 mg/kg/day liraglutide (i.h.) was administered to the Llirag and Hlirag groups, respectively. Control and diabetic groups were injected with the same volume of normal saline. Body weights and fasting blood glucose levels (Bayer Contour TS glucometer, Hamburg, Germany) were recorded monthly. After 12 weeks of treatment, an oral glucose tolerance test, in which 20% glucose was gavaged at a dose of 2 g/kg, was performed after 10 h of fasting. Tail vein blood glucose levels at 0, 30, 60, and 120 min were measured. At the end of the study, all rats were fasted for 10 h and anesthetized with ketamine (100 mg/kg i.p., Pharmacia and Upjohn Ltd, Crawley, U.K.), then blood samples were collected from the abdominal aorta. Finally, the rats were killed by decapitation. Cardiac tissue was quickly collected, frozen in liquid nitrogen, and stored at −80°C for gene microarray analysis.

### Metabolic profile analysis

Triglyceride (TG), total cholesterol (TC), high-density lipoprotein (HDL), and low-density lipoprotein (LDL) levels were measured by an enzyme end point method (Roche Diagnostics, GmbH, Mannheim, Germany). Fasting serum adiponectin and insulin levels were determined by enzyme-linked immunosorbent assay (Millipore, Billerica, MA, U.S.A.). Homeostasis model assessment of insulin resistance (HOMA-IR) scores was calculated using the following formula: FBG (mmol/l) × fasting insulin (μIU/ml)/22.5.

### Serum nitric oxide and antioxidant markers

Serum nitric oxide (NO) and GSH/GSSG levels were determined using Fluorometric Assay Kit (Cayman Chemical, Ann Arbor, MI, U.S.A.) and Thiol Green Indicator fluorometric method (Abcam, Cambridge, MA, U.S.A.), respectively.

### Tail cuff systolic blood pressure and heart rate measurements

Systolic blood pressure (SBP) and heart rate (HR) were measured by tail-cuff plethysmography (BP98A, Softron, Tokyo, Japan). After prewarming at 25°C for at least 5 min, the first five cycles were used as acclimatization cycles. After that, the mean blood pressure was recorded for the next five consecutive cycles.

### Left ventricle function measurement by echocardiography

Rats were anesthetized by inhaling 1% isoflurane with 99% O_2_. During anesthesia, left ventricle (LV) diameter was determined using a Vevo 2100 Ultrasound System (Visual Sonics, Toronto, Ontario, Canada). Fractional shortening (FS) was calculated as follow: FS% = (LVEDD – LVESD)/LVEDD × 100, where LVEDD and LVESD are the LV end-diastolic diameter and the LV end-systolic diameter, respectively [8]. Increased FS indicated better LV contractile function [9].

### RNA extraction and gene microarray hybridization

Total RNA was extracted from cardiac tissue by using a mirVana™ RNA Isolation Kit (Ambion, Sao Paulo, SP, Brazil). Total RNA was transcribed into double-stranded cDNA and then synthesized into double-stranded cRNA. The second cycle cRNA was then labeled with biotin. The biotinylated cRNA was purified, fragmented, and hybridized to an Affymetrix GeneChip Rat Gene 2.0 ST whole transcript-based array (Affymetrix Technologies, Santa Clara, CA, U.S.A.). After washing and staining, the microarrays were scanned using an Affymetrix Scanner 3000 7G (Santa Clara, CA, U.S.A.).

### Microarray bioinformatics analysis

Expression Console Software (version 1.4.1, Affymetrix, Santa Clara, CA, U.S.A.) was used to analyze the microarray signals. Differentially expressed genes were defined as having a fold change >1.5 and *P*-value <0.05 (one-way ANOVA). The raw microarray data have been submitted to the Gene Expression Omnibus (GEO) repository (GSE102194). The enrichment analysis of differentially expressed genes was performed by gene ontology (GO) and Kyoto Encyclopedia of Genes and Genomes (KEGG) pathway analysis with Database for Annotation, Visualization, and Integrated Discovery (DAVID) software (http://david.abcc.ncifcrf.gov/) [10]. The gene interaction network was drawn using String software (http://string-db.org/) [11].

### Real-time PCR

Total RNA was extracted from cardiac tissue. Reverse transcription products were tested by real-time PCR. The primers are listed in Table 1. Real-time PCR was performed on an ABI Prism 7500 Real-Time PCR System (Applied Biosystems, Foster City, CA, U.S.A.). The cycling conditions were 95°C for 10 min, followed by 40 cycles of 95°C for 15 s, and 60°C for 30 s. β-actin was used as an internal control. Samples were run in triplicate. The 2^−ΔΔ*C*^_t_ method was used to calculate the relative expression levels.

**Table 1:** Oligonucleotide sequences for qPCR analysis

Gene symbol	Genbank ID	Forward primer	Reverse primer	Product size (bp)
*Angptl3*	NM_001025065	AAAGGGTTTTGGGAGGCTTGA	CCCAAAAGCGCTATGGTCTC	117
*Dgat1*	NM_053437	GAACCGCTTCTTCCAAGGGA	AGAACTCCAGGCCCAGGTTA	177
*Dgat2*	NM_001012345	ACCTACCTCGGATCTCGACC	CTGATCCATGCCCCAGCC	105
*Ephx2*	NM_022936	CGTTCGACCTTGACGGAGTG	CTGGAAAGCGCCAAGTAGGA	107
*Nr1h3*	NM_031627	GAGTCATCCGAGCCTACAGC	AAGAATCCCTTGCAGCCCTC	191
*Pparα*	NM_013196	ATTGGCGTTCGCAGCTGTTT	CTCGTGTGCCCTCCCTCAAG	102
*Srebf1*	NM_001276707	CCATGGACGAGCTACCCTTC	GGCATCAAATAGGCCAGGGA	149
*β-actin*	NM_031144	ACTCTGTGTGGATTGGTGGC	CGCAGCTCAGTAACAGTCCG	140

Abbreviations: *Angptl3*, angiopoietin-like 3; *Dgat1*, diacylglycerol O-acyltransferase 1; *Dgat2*, diacylglycerol O-acyltransferase 2; *Ephx2*, epoxide hydrolase 2; *Nr1h3* (LXRα), nuclear receptor subfamily 1, group H, member 3; *Pparα*, peroxisome proliferator activated receptor α, *Srebf1*, sterol regulatory element binding transcription factor 1.

### Statistical analysis

GraphPad Prism software (version 5.0, San Diego, CA, U.S.A.) was used for statistical analyses. All values are shown as the mean ± S.D. Group data were analyzed using one-way ANOVA followed by Student’s *t* test. *P*<0.05 was considered to be statistically significant.

## Results

### Effect of liraglutide on body weight, serum lipid profile, blood glucose, adiponectin, and serum fasting insulin levels

**Figure 1:**
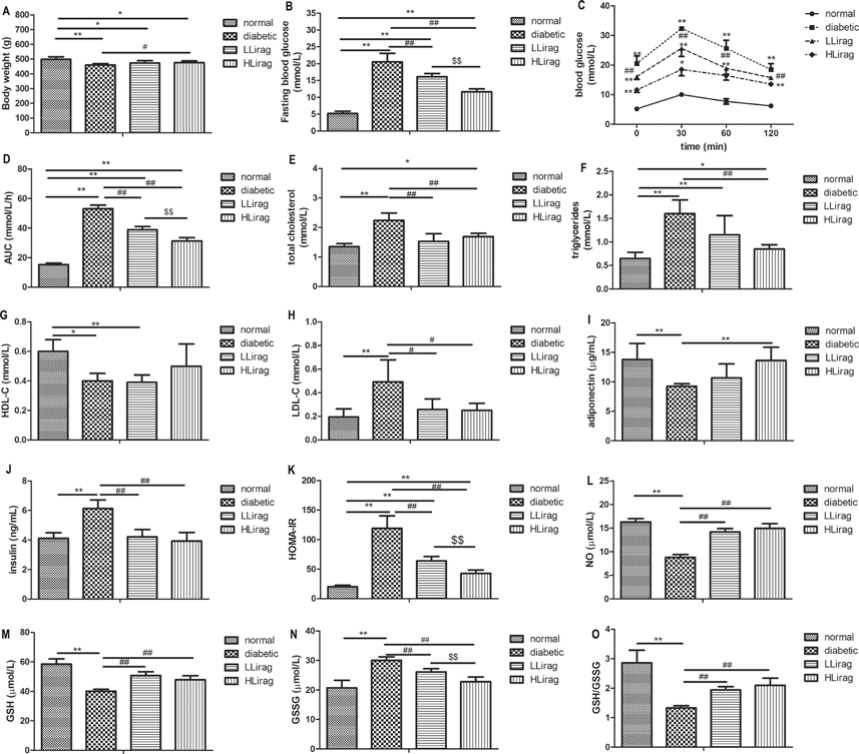
Effect of liraglutide on metabolic indexes in diabetic rats. (**A**) Body weight, (**B**) fasting blood glucose, (**C**) blood glucose in OGTT, (**D**) AUC in OGTT, (**E**) TC, (**F**) TG, (**G**) HDL, (**H**) LDL, (**I**) adiponectin, (**J**) fasting insulin, (**K**) HOMA-IR, (**L**) NO, (**M**) GSH, (**N**) GSSG, and (**O**) GSH/GSSG. Values are mean ± S.D. (*n*=6), *P<0.05, **P<0.01 compared with normal group; ^#^*P*<0.05, ^##^*P*<0.01 compared with diabetic group; ^$$^*P*<0.01 compared with Llirag group.

Liraglutide significantly reduced the body weights of diabetic rats (*P*<0.05, Figure 1A). In addition, liraglutide dose dependently reduced fasting blood glucose levels and blood glucose and area under the curve (AUC) values for oral glucose tolerance tests ( OGTTs, *P*<0.01, Figure 1B–D). The rats that underwent 12-week liraglutide treatment had lower TC (*P*<0.01, Figure 1E) and LDL-c levels (*P*<0.05, Figure 1H). Only the high dose of liraglutide reduced serum TG levels and increased serum adiponectin level in diabetic rats (*P*<0.01, Figure 1F,I). Compared with diabetic rats, liraglutide-treated rats had lower serum fasting insulin levels and HOMA-IR scores (*P*<0.01, Figure 1J,K).

### Effect of liraglutide on serum NO and antioxidant markers

Serum NO, GSH level, and GSH/GSSG ratio in diabetic rats were lower than control rats (*P*<0.01, Figure 1L,M,O). Liraglutide treatment moderated this decrease (*P*<0.01, Figure 1L,M,O). Serum GSSG level in diabetic group was higher than control rats (*P*<0.01, Figure 1N). Liraglutide reduced serum GSSG level dose independently (*P*<0.01, Figure 1N).

### Effect of liraglutide on cardiac function

**Figure 2:**
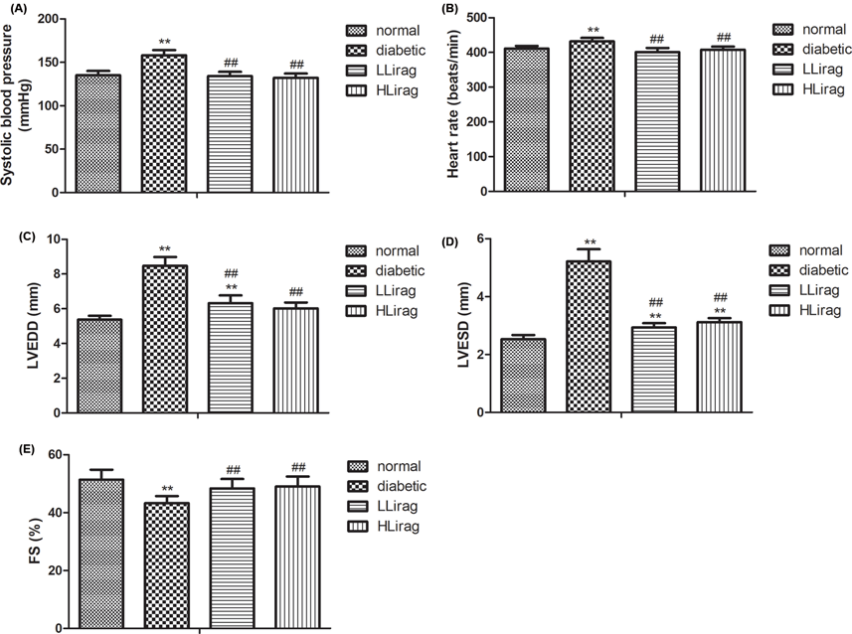
Effect of liraglutide on cardiac function in diabetic rats. (**A**) SBP, (**B**) HR, (**C**) LVEDD, (**D**) LVESD, and (**E**) FS. *n*=6. **P<0.01 compared with control; ^##^*P*<0.01 compared with diabetic.

SBP, HR, LVEDD, and LVESD levels in the diabetic group were significantly increased (*P*<0.01, Figure 2A–D). However, diabetic rats had lower %FS values than normal control rats (*P*<0.01, Figure 2E). Liraglutide treatment decreased SBP, HR, LVEDD, and LVESD levels and increased % FS values (*P*<0.01, Figure 2A–E). These results suggest that liraglutide moderated LV dysfunction and decreased blood pressure and HRs.

### Microarray data analysis of Hlirag and diabetic groups

A total of 269 differentially expressed genes were screened out from Hlirag group (fold change >1.5, *P*<0.05); these included 166 up-regulated genes and 105 down-regulated genes. The differentially expressed genes were enriched in 11 pathways (*P*<0.001, Table 2). The top five pathways were cardiac muscle contraction, non-alcoholic fatty liver disease (NAFLD), oxidative phosphorylation, metabolic pathways, metabolic pathways, and Alzheimer’s disease. The significant biological processes (BPs) in the GO categories are listed in Table 3 (*P*<0.01). The top ten BP terms were hydrogen ion transmembrane transport, cholesterol homeostasis, lipid homeostasis, positive regulation of TG biosynthetic process, fatty acid β-oxidation using acyl-CoA dehydrogenase, fatty acid β-oxidation, response to cAMP, very long-chain fatty acid catabolic process, circadian rhythm, and lipid metabolic process.

**Table 2:** The enriched KEGG pathway with differentially expressed genes (*P*<0.001)

Pathway ID	Pathway name	Count	Fold enrichment	*P*-value
rno04932	NAFLD	14	6.003	4.39 × 10^−7^
rno04260	Cardiac muscle contraction	10	8.905	1.42 × 10^−6^
rno01100	Metabolic pathways	39	2.095	4.16 × 10^−6^
rno00190	Oxidative phosphorylation	12	5.7889	5.79 × 10^−6^
rno05012	Parkinson’s disease	12	5.484	9.74 × 10^−6^
rno05010	Alzheimer’s disease	13	4.961	9.80 × 10^−6^
rno00640	Propanoate metabolism	6	14.885	4.09 × 10^−5^
rno01200	Carbon metabolism	10	5.740	5.27 × 10^−5^
rno00280	Valine, leucine, and isoleucine degradation	7	8.840	1.21 × 10^−4^
rno05016	Huntington’s disease	12	4.106	1.41× 10^−4^
rno01130	Biosynthesis of antibiotics	12	3.806	2.73 × 10^−4^

**Table 3:** The enriched GO terms with differentially expressed genes (*P*<0.01)

Term ID	Term name	Count	*P*-value	Fold enrichment	Catalog
GO:1902600	Hydrogen ion transmembrane transport	8	1.221 × 10^−5^	0.0193	BP
GO:0042632	Cholesterol homeostasis	7	0.000124	0.197	BP
GO:0055088	Lipid homeostasis	6	0.000148	0.236	BP
GO:0010867	Positive regulation of triglyceride biosynthetic process	4	0.00105	1.669	BP
GO:0033539	Fatty acid β-oxidation using acyl-CoA dehydrogenase	4	0.00148	2.329	BP
GO:0006635	Fatty acid β-oxidation	5	0.00231	3.616	BP
GO:0051591	Response to cAMP	6	0.00245	3.825	BP
GO:0042760	Very long-chain fatty acid catabolic process	3	0.00296	4.601	BP
GO:0007623	Circadian rhythm	7	0.00361	5.589	BP
GO:0006629	Lipid metabolic process	6	0.00458	7.039	BP
GO:0090181	Regulation of cholesterol metabolic process	3	0.00499	7.647	BP
GO:0043401	Steroid hormone mediated signaling pathway	5	0.00538	8.209	BP
GO:0006631	Fatty acid metabolic process	5	0.00606	9.213	BP
GO:0019217	Regulation of fatty acid metabolic process	3	0.00619	9.399	BP
GO:2000188	Regulation of cholesterol homeostasis	3	0.00619	9.399	BP
GO:0030522	Intracellular receptor signaling pathway	4	0.00737	11.085	BP
GO:0019432	Triglyceride biosynthetic process	3	0.00894	13.296	BP
GO:0006366	Transcription from RNA polymerase II promoter	12	0.00976	14.419	BP
GO:0005739	Mitochondrion	43	1.503 × 10^−6^	2.199	Cellular components
GO:0005743	Mitochondrial inner membrane	13	0.000465	3.403	Cellular components
GO:0005777	Peroxisome	8	0.000731	5.373	Cellular components
GO:0005746	Mitochondrial respiratory chain	3	0.00620	24.584	Cellular components
GO:0004129	Cytochrome *c* oxidase activity	6	4.237 × 10^−5^	15.081	Molecular function
GO:0003700	Transcription factor activity, sequence-specific DNA binding	22	0.000415	2.359	Molecular function
GO:0001077	Transcriptional activator activity, RNA polymerase II core promoter proximal region sequence-specific binding	11	0.00127	3.483	Molecular function
GO:0000978	RNA polymerase II core promoter proximal region sequence-specific DNA binding	12	0.00397	2.789	Molecular function
GO:0043565	Sequence-specific DNA binding	16	0.00479	2.265	Molecular function
GO:0009055	Electron carrier activity	5	0.00505	7.181	Molecular function
GO:0003707	Steroid hormone receptor activity	5	0.00505	7.181	Molecular function
GO:0004879	RNA polymerase II transcription factor activity, ligand-activated sequence-specific DNA binding	4	0.00912	9.192	Molecular function
GO:0046982	Protein heterodimerization activity	15	0.00946	2.170	Molecular function
GO:0044212	Transcription regulatory region DNA binding	9	0.00950	3.054	Molecular function

**Figure 3:**
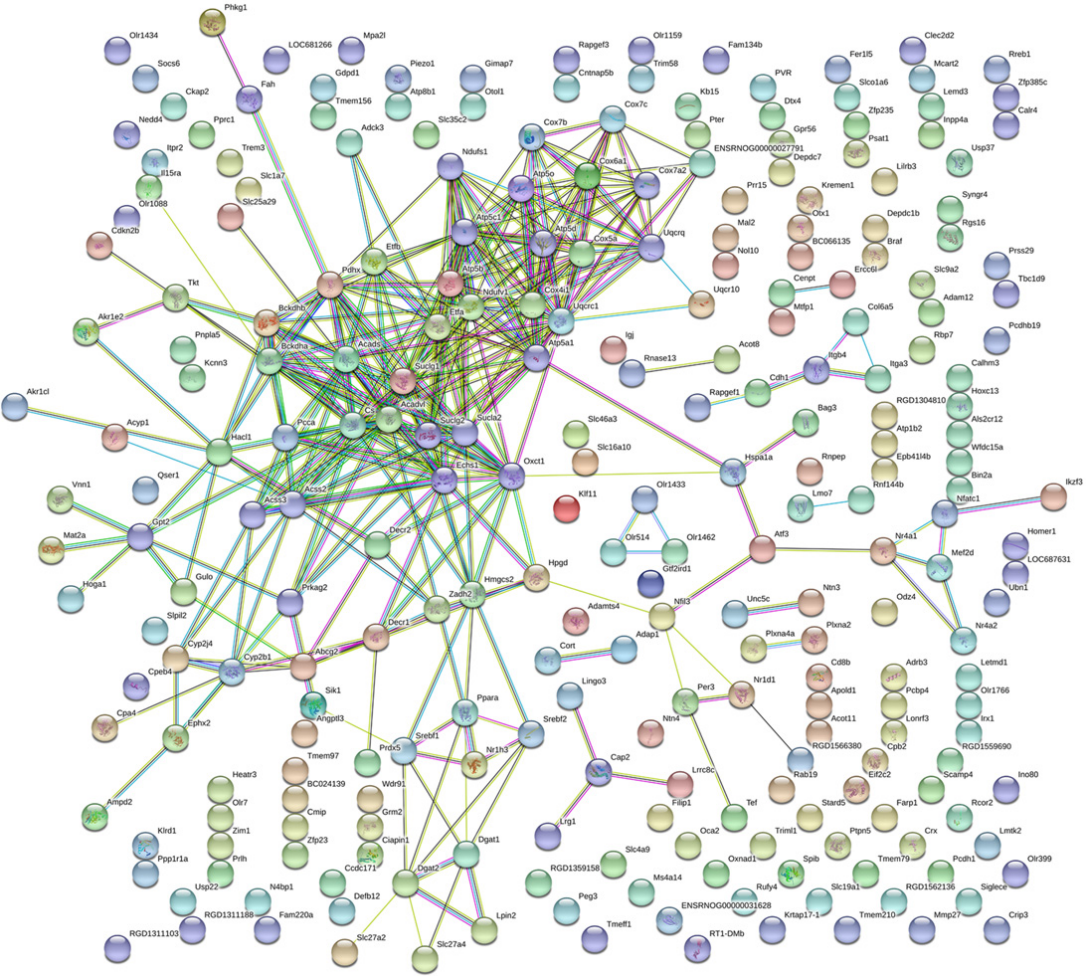
Protein–protein interaction network in Hlirag group compared with diabetic group The nods stand for differentially expressed genes in Hlirag group compared with diabetic group. The lines stand for the interactions between two proteins.

All the 271 differentially expressed genes were mapped using String online software. The results showed that there were 253 interactions with a total of 256 joint edges (Figure 3). Twenty-one nodes had more than ten joint edges. These nodes involved 137 joint edges. These 21 genes are listed in Table 4. The top ten genes were citrate synthase (*Cs*), ubiquinol-cytochrome *c* reductase core protein I (*Uqcrc1*), acyl-CoA dehydrogenase, very long chain (*Acadv1*), 3-oxoacid CoA transferase 1 (*Oxct1*), NADH dehydrogenase (ubiquinone) flavoprotein 1 (*Ndufv1*), succinate-CoA ligase, alpha subunit (*Suclg1*), enoyl CoA hydratase, short chain, 1, mitochondrial (*Echs1*), acyl-CoA dehydrogenase, C2–C3 short chain (*Acads*), and branched chain ketoacid dehydrogenase E1, α-polypeptide (*Bckdha*).

**Table 4:** A list of genes with connective degree more than ten in the String network

Gene accession	Gene symbol	Gene name	Degree
NM_130755	*Cs*	Citrate synthase	20
NM_001004250	*Uqcrc1*	Ubiquinol-cytochrome *c* reductase core protein I	18
NM_012891	*Acadvl*	Acyl-CoA dehydrogenase, very long chain	17
NM_001127580	*Oxct1*	3-Oxoacid CoA transferase 1	16
NM_001006972	*Ndufv1*	NADH dehydrogenase (ubiquinone) flavoprotein 1	15
NM_053752	*Suclg1*	Succinate-CoA ligase, alpha subunit	15
NM_078623	*Echs1*	Enoyl CoA hydratase, short chain, 1, mitochondrial	14
NM_022512	*Acads*	Acyl-CoA dehydrogenase, C-2 to C-3 short chain	13
NM_012782	*Bckdha*	Branched chain ketoacid dehydrogenase E1, α-polypeptide	13
NM_134364	*Atp5b*	ATP synthase, H^+^ transporting, mitochondrial F1 complex, β-polypeptide	12
NM_017202	*Cox4i1*	Cytochrome *c* oxidase subunit IV isoform 1	12
NM_145783	*Cox5a*	Cytochrome *c* oxidase, subunit Va	12
NM_001107793	*Acss2*	Acyl-CoA synthetase short-chain family member 2	11
NM_138883	*Atp5o*	ATP synthase, H^+^ transporting, mitochondrial F1 complex, O subunit	11
ENSRNOT00000077826	*Decr2*	2,4-Dienoyl CoA reductase 2, peroxisomal	11
NM_001044242	*Pdhx*	Pyruvate dehydrogenase complex, component X	11
NM_001108387	*Sucla2*	Succinate-CoA ligase, ADP-forming, β-subunit	11
NM_001025134	*Uqcrq*	Ubiquinol-cytochrome *c* reductase, complex III subunit VII	11
NM_019267	*Bckdhb*	Branched chain keto acid dehydrogenase E1, β-polypeptide	10
NM_012814	*Cox6a1*	Cytochrome *c* oxidase, subunit VIa, polypeptide 1	10
NM_053493	*Hacl1*	2-Hydroxyacyl-CoA lyase 1	10

### Confirmation by quantitative Polymerase Chain Reaction (qPCR)

**Figure 4:**
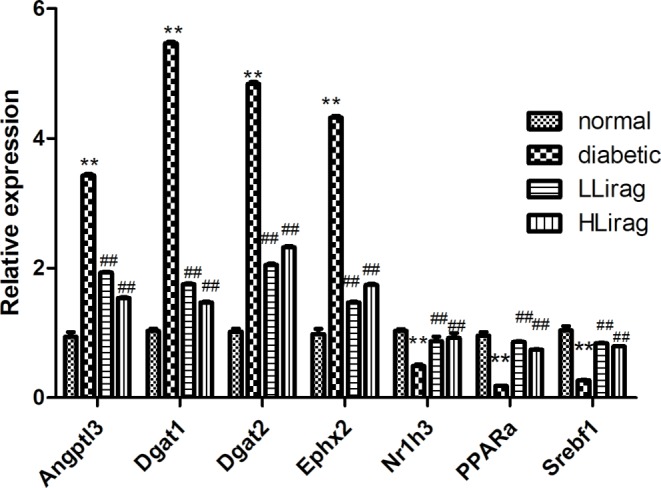
Confirmation of five representative differentially expressed genes by qPCR Values are mean ± S.D. (*n*=6), **P<0.01 compared with normal group; ^##^*P*<0.01 compared with diabetic group.

To validate the microarray results, we analyzed the mRNA expression levels of representative gene by using qPCR. As shown in Figure 4, the relative mRNA levels of LXRα, nuclear receptor subfamily 1, group H, member 3 (*Nr1h3*), sterol regulatory element binding transcription factor 1 (*Srebf1*) and peroxisome proliferator activated receptor α (*PPARα*) in the Hlirag group were significantly higher, whereas the mRNA levels of angiopoietin-like 3 (*Angptl3*), diacylglycerol O-acyltransferase 1 (*Dgat1*), diacylglycerol O-acyltransferase 2 (*Dgat2*), and epoxide hydrolase 2 (*Ephx2*) were lower than those in diabetic rat group (*P*<0.01). These outcomes were consistent with the microarray results.

## Discussion

In the present study, as expected, liraglutide reduced blood glucose levels and moderated insulin resistance in diabetic rats. Moreover, we found that liraglutide reduced the body weights in diabetic rats. Liraglutide-treated rats also had lower TC and LDL-c levels and higher adiponectin levels. Clinical trials proved that liraglutide was effective at reducing blood glucose levels and body weights [12–14]. Liraglutide at 3.0 mg/day has been approved by the U.S. Food and Drug Administration (FDA) for treating obesity since 2014. In human studies, liraglutide treatment at 3.0 mg for 56 weeks decreased insulin resistance [15]. In addition, liraglutide treatment dose dependently increased plasma adiponectin in Chinese type 2 diabetes [16].

Regarding cardiac function, we found that liraglutide reduced SBP, HR, LVEDD, and LVESD levels, increased %FS values and serum NO levels in diabetic rats. These results indicate the beneficial effects of liraglutide on cardiac function. HFD-fed mice have cardiac ceramide accumulation. One week of liraglutide treatment improved cardiac ER homeostasis and cardiac function [17]. A clinical trial revealed that liraglutide reduced LVEF in patients with heart failure (HF) [18]. Interestingly, liraglutide reduced both systolic and diastolic blood pressure in hypertensive mice through the atrial natriuretic peptide (ANP) axis [19]. In a meta-analysis of 16 randomized controlled trials, GLP-1 receptor agonists (exenatide and liraglutide) reduced systolic pressure (SBP) and diastole pressure (DBP) by 1–5 mmHg compared with other antidiabetic drugs in diabetic patients [20]. Reductions in blood pressure were not related to weight loss or hemoglobin A1c (HbA1c) improvement [21].

We found that liraglutide increased PPARα expression in the cardiac tissue of diabetic rats. PPARs have three forms: α, γ, and δ. They can bind with retinoid X receptor (RXR) to regulate energy utilization and storage [22]. Recent results implicate PPARs in the regulation of inflammation and atherosclerosis [23]. In the heart, both PPARα and PPARδ can regulate lipid metabolism. In addition to lipid metabolism, PPAR-γ also modulates glucose metabolism [24–26]. Previous studies found that PPAR-α expression is down-regulated in diabetic rat hearts [27–30]. Many studies indicate that oxidative stress increases in diabetic status [31,32] and contributes to inhibition of PPAR-α in cardiomyocytes [33]. Our data also showed that diabetic rats had lower GSH/GSSG ratio, and liraglutide treatment increased serum GSH/GSSG ratio. GSH/GSSG ratio is an important antioxidant biomarker [34].

Interestingly, in our study, liraglutide reduced *Dgat1* and *Dgat2* expression in the hearts of diabetic rats. DGAT has two isoforms: DGAT1 and DGAT2. They are the enzymes that catalyze the final step in the biosynthesis of TG [35]. DGAT is the target gene of PPARα. DGAT2 appears to be a key enzyme that controls TG homeostasis *in vivo* and regulates fatty acid storage [36]. A previous study found that DGAT1 and DGAT2 expression was increased in diabetic rat hearts [37]. Increased DGAT expression generated reactive oxidative stress, and caused myocardial damage in DM cardiomyopathy [38]. Thus, our results indicate that liraglutide reverses oxidative stress generated by diabetic status to increase PPARα expression, leads to reduce the expression of DGAT, and also finally inhibits reactive oxidative stress.

Our research found that liraglutide increased *Nr1h3* (LXRα) and *Srebf1* expression in diabetic rat hearts. LXRs have important role in the regulation of cholesterol and fatty acid metabolism. It forms heterodimer with RXR [39]. *Srebf* is directly induced by LXRs through an RXR/LXR-binding site on the *Srebf* gene promoter [40,41]. SREBF is a transcription factor that regulates lipogenic enzymes by binding to sterol response elements [42]. Thus, our data supports that liraglutide treatment activates cardiac LXRα and *Srebf1* expression in diabetic rats.

We also found that liraglutide reduced *Ephx2* expression in diabetic rat hearts. Soluble epoxide hydrolase (sEH) is an *Ephx2* gene product. Arachidonic acid (AA) can be transferred to epoxyeicosatrienoic acids (EETs) by cytochrome P-450 (CYP) epoxygenases. EETs are signaling molecules that regulate blood pressure [43–47], inflammation [44,45,47,48], and glucose homeostasis [49,50]. However, EETs have a short half-life and may be metabolized by sEH into dihydroxyeicosatrienoic acids (DHETs) with relatively weak activity. Hearts in sEH null mice had improved post-ischemic recovery of Lv developed pressure (LVDP), reduced infarct size after ischemia and reperfusion [51], and reduced survival after cardiac arrest and cardiopulmonary resuscitation [52]. Therefore, sEH is a promising target for treating cardiovascular disease. sEH inhibitors have been indicated as beneficial treatments in animal models of high blood pressure [43,44,46], inflammation [44,48,53], myocardial injury [44,54–56], ischemia–reperfusion [57,58], pathological cardiac hypertrophy [44], and insulin resistance [49,50] and have been shown to protect heart structure and function [59]. Our study provided evidence that as a sEH ingibitor, liraglutide can preserve cardiac function.

We also found that liraglutide reduced *Angptl3* expression in diabetic rat hearts. Lipoprotein lipase (LPL) metabolized TG into free fatty acids (FFAs). LPL overexpression is correlated with reduced plasma TG levels and decreased cardiovascular risks [60]. However, LPL null models have severe hypertriglyceridemia [60]. The angiopoietin-like protein (ANGPTL) family is a key regulator of LPL [61,62]. ANGPTL3 is an endogenous inhibitor of LPL. Rare loss-of-function variants for ANGPTL3 have been associated with decreased TG levels as well as decreased low-density lipoprotein-cholesterol (LDL-c) and high-density lipoprotein-cholesterol (HDL-c) levels in family and general population studies in humans [62–70]. In addition, these subjects demonstrate an absence of coronary atherosclerotic plaques [71]. Another human population study showed that plasma ANGPTL3 levels were increased in myocardial infarction patients [71]. Heterozygous carries of ANGPTL3 loss-of-function mutations had a 34% reduction in CAD risk [71]. *Angptl3* deletion was also reported to reduce the development of atherosclerosis in apolipoprotein E (apoE)-deficient mice [72]. Recently, a human monoclonal antibody against Angptl3 in dyslipidemic mice and against ANGPTL3 in healthy human subjects with elevated levels of TGs or LDL-c significantly reduced serum TG, HDL-c, and LDL-c levels and decreased the odds of atherosclerotic cardiovascular disease [73]. Another research group showed that treating mice and human subjects with antisense oligonucleotides targeting Angptl3 messenger RNA reduced atherogenic lipoproteins and retarded the progression of atherosclerosis [74].

## Conclusion

**Figure 5:**
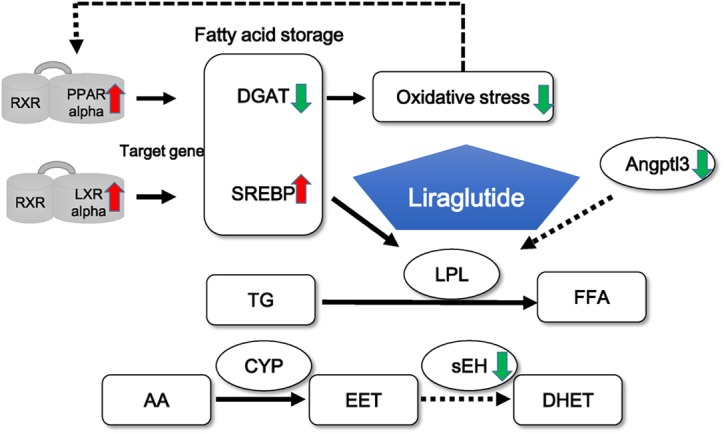
Liraglutide activates PPARα, which binds to RXR Then PPARα inhibits its target gene, *DGAT* to inhibit oxidative stress. Liraglutide also activates *Nr1h3* and SREBP and inhibits Angptl3 to activate LPL, leading the production of FFAs. Moreover, liraglutide inhibits sEH expression to increase EET.

In conclusion, liraglutide prevents cardiac dysfunction by activating cardiac PPARα to inhibit *Dgat* expression and oxidative stress in diabetic rats (Figure 5). The present study provides a potential mechanism for the protective cardiac effects of a GLP-1 analog in a model of diabetes.

## Abbreviations

ANGPTL3: angiopoietin-like 3
AMPK: activated protein kinase
apoE: apolipoprotein E
AUC: area uncer the curve
BP: biological process
CAD: coronary artery disease
DBP: diastole pressure
DCM: diabetic cardiomyopathy
DGAT: diacylglycerol O-acyltransferase
EET: epoxyeicosatrienoic acid
ER: endoplasmic reticulum
FS: fractional shortening
GLP-1: glucagon-like peptide-1
GLP-1R: GLP-1 receptor
GO: gene ontology
HbA1c: hemoglobin A1c
HDL-c: high-density lipoprotein-cholesterol
HFD: high-fat diet
Hlirag: high-dose liraglutide
HOMA-IR: homeostasis model assessment of insulin resistance
HR: heart rate
KEGG: Kyoto Encyclopedia of Genes and Genomes
LDL-c: low-density lipoprotein-cholesterol
Llirag: low-dose liraglutide
LV: left ventricular
LVEDD: LV end-diastolic diameter
LVEF: LV ejection fraction
LVESD: LV end-systolic diameter
OGTT: oral glucose tolerance test
PPAR: peroxisome proliferator activated receptor
qPCR: quantitative polymerase chain reaction
RXR: retinoid X receptor
SBP: systolic blood pressure
sEH: soluble epoxide hydrolase
SBP: systolic pressure
Sirt1: sirtuin 1
STZ: streptozotocin
TC: total cholesterol
TG: triglyceride

## References

[1] Ogurtsova K., da Rocha Fernandes J.D., Huang Y., Linnenkamp U., Guariguata L., Cho N.H. (2017) IDF Diabetes Atlas: global estimates for the prevalence of diabetes for 2015 and 2040. Diabetes Res. Clin. Pract. 128, 40–50 10.1016/j.diabres.2017.03.024 28437734

[2] Capes S.E., Hunt D., Malmberg K. and Gerstein H.C. (2000) Stress hyperglycaemia and increased risk of death after myocardial infarction in patients with and without diabetes: a systematic overview. Lancet 355, 773–778 10.1016/S0140-6736(99)08415-9 10711923

[3] Jia G., DeMarco V.G. and Sowers J.R. (2016) Insulin resistance and hyperinsulinaemia in diabetic cardiomyopathy. Nat. Rev. Endocrinol. 12, 144–153 10.1038/nrendo.2015.216 26678809 PMC4753054

[4] Jia G., Whaley-Connell A. and Sowers J.R. (2017) Diabetic cardiomyopathy: a hyperglycaemia- and insulin-resistance-induced heart disease. Diabetologia, 10.1007/s00125-017-4390-4 28776083 PMC5720913

[5] Cardillo C. (2013) Drug treatments to restore vascular function and diabesity. Ann. Pharm. Fr. 71, 27–33 10.1016/j.pharma.2012.09.001 23348853

[6] Chen W.R., Hu S.Y., Chen Y.D., Zhang Y., Qian G., Wang J. (2015) Effects of liraglutide on left ventricular function in patients with ST-segment elevation myocardial infarction undergoing primary percutaneous coronary intervention. Am. Heart J. 170, 845–854 10.1016/j.ahj.2015.07.014 26542491

[7] Inoue T., Inoguchi T., Sonoda N., Hendarto H., Makimura H., Sasaki S. (2015) GLP-1 analog liraglutide protects against cardiac steatosis, oxidative stress and apoptosis in streptozotocin-induced diabetic rats. Atherosclerosis 240, 250–259 10.1016/j.atherosclerosis.2015.03.026 25818251

[8] Derumeaux G., Mulder P., Richard V., Chagraoui A., Nafeh C., Bauer F. (2002) Tissue doppler imaging differentiates physiological from pathological pressure-overload left ventricular hypertrophy in rats. Circulation 105, 1602–1608 10.1161/01.CIR.0000012943.91101.D7 11927530

[9] Apaijai N., Pintana H., Chattipakorn S.C. and Chattipakorn N. (2012) Cardioprotective effects of metformin and vildagliptin in adult rats with insulin resistance induced by a high-fat diet. Endocrinology 153, 3878–3885 10.1210/en.2012-1262 22621958

[10] Dennis G. Jr, Sherman B.T., Hosack D.A., Yang J., Gao W., Lane H.C. (2003) DAVID: Database for Annotation, Visualization, and Integrated Discovery. Genome Biol. 4, P3 10.1186/gb-2003-4-5-p3 12734009 PMC193660

[11] Szklarczyk D., Franceschini A., Wyder S., Forslund K., Heller D., Huerta-Cepas J. (2015) STRING v10: protein–protein interaction networks, integrated over the tree of life. Nucleic Acids Res. 43, D447–D452 10.1093/nar/gku1003 25352553 PMC4383874

[12] Sun F., Chai S., Li L., Yu K., Yang Z., Wu S. (2015) Effects of glucagon-like peptide-1 receptor agonists on weight loss in patients with type 2 diabetes: a systematic review and network meta-analysis. J. Diabetes Res. 2015, 157201 10.1155/2015/157201 25688373 PMC4320855

[13] Harris K.B. and McCarty D.J. (2015) Efficacy and tolerability of glucagon-like peptide-1 receptor agonists in patients with type 2 diabetes mellitus. Ther. Adv. Endocrinol. Metab. 6, 3–18 10.1177/2042018814558242 25678952 PMC4321868

[14] Amori R.E., Lau J. and Pittas A.G. (2007) Efficacy and safety of incretin therapy in type 2 diabetes: systematic review and meta-analysis. JAMA 298, 194–206 10.1001/jama.298.2.194 17622601

[15] Christou G.A., Katsiki N. and Kiortsis D.N. (2016) The current role of liraglutide in the pharmacotherapy of obesity. Curr. Vasc. Pharmacol. 14, 201–207 10.2174/1570161113666150615111951 26074046

[16] Li D., Xu X., Zhang Y., Zhu J., Ye L., Lee K.O. (2015) Liraglutide treatment causes upregulation of adiponectin and downregulation of resistin in Chinese type 2 diabetes. Diabetes Res. Clin. Pract. 110, 224–228 10.1016/j.diabres.2015.05.051 26376464

[17] Noyan-Ashraf M.H., Shikatani E.A., Schuiki I., Mukovozov I., Wu J., Li R.K. (2013) A glucagon-like peptide-1 analog reverses the molecular pathology and cardiac dysfunction of a mouse model of obesity. Circulation 127, 74–85 10.1161/CIRCULATIONAHA.112.091215 23186644

[18] Moberly S.P., Mather K.J., Berwick Z.C., Owen M.K., Goodwill A.G., Casalini E.D. (2013) Impaired cardiometabolic responses to glucagon-like peptide 1 in obesity and type 2 diabetes mellitus. Basic Res. Cardiol. 108, 365 10.1007/s00395-013-0365-x 23764734 PMC3731771

[19] Kim M., Platt M.J., Shibasaki T., Quaggin S.E., Backx P.H., Seino S. (2013) GLP-1 receptor activation and Epac2 link atrial natriuretic peptide secretion to control of blood pressure. Nat. Med. 19, 567–575 10.1038/nm.3128 23542788

[20] Wang B., Zhong J., Lin H., Zhao Z., Yan Z., He H. (2013) Blood pressure-lowering effects of GLP-1 receptor agonists exenatide and liraglutide: a meta-analysis of clinical trials. Diabetes Obes. Metab. 15, 737–749 10.1111/dom.12085 23433305

[21] Katout M., Zhu H., Rutsky J., Shah P., Brook R.D., Zhong J. (2014) Effect of GLP-1 mimetics on blood pressure and relationship to weight loss and glycemia lowering: results of a systematic meta-analysis and meta-regression. Am. J. Hypertens. 27, 130–139 10.1093/ajh/hpt196 24263424

[22] Chen L., Jia Z. and Yang G. (2014) PPARs and metabolic syndrome. PPAR Res. 2014, 832606 10.1155/2014/832606 24782893 PMC3982464

[23] Brown J.D. and Plutzky J. (2007) Peroxisome proliferator-activated receptors as transcriptional nodal points and therapeutic targets. Circulation 115, 518–533 10.1161/CIRCULATIONAHA.104.475673 17261671

[24] Finck B.N. (2007) The PPAR regulatory system in cardiac physiology and disease. Cardiovasc. Res. 73, 269–277 10.1016/j.cardiores.2006.08.023 17010956

[25] Yang Q. and Li Y. (2007) Roles of PPARs on regulating myocardial energy and lipid homeostasis. J. Mol. Med. (Berl.) 85, 697–706 10.1007/s00109-007-0170-9 17356846

[26] Madrazo J.A. and Kelly D.P. (2008) The PPAR trio: regulators of myocardial energy metabolism in health and disease. J. Mol. Cell Cardiol. 44, 968–975 10.1016/j.yjmcc.2008.03.021 18462747

[27] Lee T.I., Kao Y.H., Chen Y.C., Pan N.H. and Chen Y.J. (2010) Oxidative stress and inflammation modulate peroxisome proliferator-activated receptors with regional discrepancy in diabetic heart. Eur. J. Clin. Invest. 40, 692–699 10.1111/j.1365-2362.2010.02318.x 20561028

[28] Depre C., Young M.E., Ying J., Ahuja H.S., Han Q., Garza N. (2000) Streptozotocin-induced changes in cardiac gene expression in the absence of severe contractile dysfunction. J. Mol. Cell Cardiol. 32, 985–996 10.1006/jmcc.2000.1139 10888252

[29] Young M.E., Patil S., Ying J., Depre C., Ahuja H.S., Shipley G.L. (2001) Uncoupling protein 3 transcription is regulated by peroxisome proliferator-activated receptor (alpha) in the adult rodent heart. FASEB J. 15, 833–845 10.1096/fj.00-0351com 11259402

[30] Yu B.C., Chang C.K., Ou H.Y., Cheng K.C. and Cheng J.T. (2008) Decrease of peroxisome proliferator-activated receptor delta expression in cardiomyopathy of streptozotocin-induced diabetic rats. Cardiovasc. Res. 80, 78–87 10.1093/cvr/cvn172 18573863

[31] Cai L., Wang Y., Zhou G., Chen T., Song Y., Li X. (2006) Attenuation by metallothionein of early cardiac cell death via suppression of mitochondrial oxidative stress results in a prevention of diabetic cardiomyopathy. J. Am. Coll. Cardiol. 48, 1688–1697 10.1016/j.jacc.2006.07.022 17045908

[32] Ye G., Metreveli N.S., Donthi R.V., Xia S., Xu M., Carlson E.C. (2004) Catalase protects cardiomyocyte function in models of type 1 and type 2 diabetes. Diabetes 53, 1336–1343 10.2337/diabetes.53.5.1336 15111504

[33] Lee T.I., Kao Y.H., Chen Y.C. and Chen Y.J. (2009) Proinflammatory cytokine and ligands modulate cardiac peroxisome proliferator-activated receptors. Eur. J. Clin. Invest. 39, 23–30 10.1111/j.1365-2362.2008.02062.x 19067734

[34] Sentellas S., Morales-Ibanez O., Zanuy M. and Alberti J.J. (2014) GSSG/GSH ratios in cryopreserved rat and human hepatocytes as a biomarker for drug induced oxidative stress. Toxicol. In Vitro 28, 1006–1015 10.1016/j.tiv.2014.04.017 24809893

[35] Shi Y. and Cheng D. (2009) Beyond triglyceride synthesis: the dynamic functional roles of MGAT and DGAT enzymes in energy metabolism. Am. J. Physiol. Endocrinol. Metab. 297, E10–E18 10.1152/ajpendo.90949.2008 19116371 PMC3735925

[36] Stone S.J., Myers H.M., Watkins S.M., Brown B.E., Feingold K.R., Elias P.M. (2004) Lipopenia and skin barrier abnormalities in DGAT2-deficient mice. J. Biol. Chem. 279, 11767–11776 10.1074/jbc.M311000200 14668353

[37] Lee T.I., Kao Y.H., Tsai W.C., Chung C.C., Chen Y.C. and Chen Y.J. (2016) HDAC inhibition modulates cardiac PPARs and fatty acid metabolism in diabetic cardiomyopathy. PPAR Res. 2016, 5938740 10.1155/2016/5938740 27446205 PMC4944062

[38] Zhang J., Xue J., Wang H., Zhang Y. and Xie M. (2011) Osthole improves alcohol-induced fatty liver in mice by reduction of hepatic oxidative stress. Phytother. Res. 25, 638–643 10.1002/ptr.3315 20981870

[39] Joseph S.B. and Tontonoz P. (2003) LXRs: new therapeutic targets in atherosclerosis? Curr. Opin. Pharmacol. 3, 192–197 10.1016/S1471-4892(03)00009-2 12681243

[40] Repa J.J., Liang G., Ou J., Bashmakov Y., Lobaccaro J.M., Shimomura I. (2000) Regulation of mouse sterol regulatory element-binding protein-1c gene (SREBP-1c) by oxysterol receptors, LXRalpha and LXRbeta. Genes Dev. 14, 2819–2830 10.1101/gad.844900 11090130 PMC317055

[41] Yoshikawa T., Shimano H., Amemiya-Kudo M., Yahagi N., Hasty A.H., Matsuzaka T. (2001) Identification of liver X receptor-retinoid X receptor as an activator of the sterol regulatory element-binding protein 1c gene promoter. Mol. Cell. Biol. 21, 2991–3000 10.1128/MCB.21.9.2991-3000.2001 11287605 PMC86928

[42] Beltowski J. (2008) Liver X receptors (LXR) as therapeutic targets in dyslipidemia. Cardiovasc. Ther. 26, 297–316 10.1111/j.1755-5922.2008.00062.x 19035881

[43] Imig J.D. (2005) Epoxide hydrolase and epoxygenase metabolites as therapeutic targets for renal diseases. Am. J. Physiol. Renal Physiol. 289, F496–F503 10.1152/ajprenal.00350.2004 16093425

[44] Imig J.D. and Hammock B.D. (2009) Soluble epoxide hydrolase as a therapeutic target for cardiovascular diseases. Nat. Rev. Drug Discov. 8, 794–805 10.1038/nrd2875 19794443 PMC3021468

[45] Inceoglu B., Schmelzer K.R., Morisseau C., Jinks S.L. and Hammock B.D. (2007) Soluble epoxide hydrolase inhibition reveals novel biological functions of epoxyeicosatrienoic acids (EETs). Prostaglandins Other Lipid Mediat. 82, 42–49 10.1016/j.prostaglandins.2006.05.004 17164131 PMC1904338

[46] Jiang H., Quilley J., Doumad A.B., Zhu A.G., Falck J.R., Hammock B.D. (2011) Increases in plasma trans-EETs and blood pressure reduction in spontaneously hypertensive rats. Am. J. Physiol. Heart Circ. Physiol. 300, H1990–H1996 10.1152/ajpheart.01267.2010 21398593 PMC3119086

[47] Spector A.A., Fang X., Snyder G.D. and Weintraub N.L. (2004) Epoxyeicosatrienoic acids (EETs): metabolism and biochemical function. Prog. Lipid Res. 43, 55–90 10.1016/S0163-7827(03)00049-3 14636671

[48] Fleming I. (2007) DiscrEET regulators of homeostasis: epoxyeicosatrienoic acids, cytochrome P450 epoxygenases and vascular inflammation. Trends Pharmacol. Sci. 28, 448–452 10.1016/j.tips.2007.08.002 17764757

[49] Luo P., Chang H.H., Zhou Y., Zhang S., Hwang S.H., Morisseau C. (2010) Inhibition or deletion of soluble epoxide hydrolase prevents hyperglycemia, promotes insulin secretion, and reduces islet apoptosis. J. Pharmacol. Exp. Ther. 334, 430–438 10.1124/jpet.110.167544 20439437 PMC2913776

[50] Luria A., Bettaieb A., Xi Y., Shieh G.J., Liu H.C., Inoue H. (2011) Soluble epoxide hydrolase deficiency alters pancreatic islet size and improves glucose homeostasis in a model of insulin resistance. Proc. Natl. Acad. Sci. U.S.A. 108, 9038–9043 10.1073/pnas.1103482108 21571638 PMC3107315

[51] Seubert J.M., Sinal C.J., Graves J., DeGraff L.M., Bradbury J.A., Lee C.R. (2006) Role of soluble epoxide hydrolase in postischemic recovery of heart contractile function. Circ. Res. 99, 442–450 10.1161/01.RES.0000237390.92932.37 16857962 PMC2072806

[52] Hutchens M.P., Nakano T., Dunlap J., Traystman R.J., Hurn P.D. and Alkayed N.J. (2008) Soluble epoxide hydrolase gene deletion reduces survival after cardiac arrest and cardiopulmonary resuscitation. Resuscitation 76, 89–94 10.1016/j.resuscitation.2007.06.031 17728042 PMC2585367

[53] Campbell W.B., Gebremedhin D., Pratt P.F. and Harder D.R. (1996) Identification of epoxyeicosatrienoic acids as endothelium-derived hyperpolarizing factors. Circ. Res. 78, 415–423 10.1161/01.RES.78.3.415 8593700

[54] Aboutabl M.E., Zordoky B.N., Hammock B.D. and El-Kadi A.O. (2011) Inhibition of soluble epoxide hydrolase confers cardioprotection and prevents cardiac cytochrome P450 induction by benzo(a)pyrene. J. Cardiovasc. Pharmacol. 57, 273–281 10.1097/FJC.0b013e3182055baf 21383588

[55] Gross G.J., Gauthier K.M., Moore J., Falck J.R., Hammock B.D., Campbell W.B. (2008) Effects of the selective EET antagonist, 14,15-EEZE, on cardioprotection produced by exogenous or endogenous EETs in the canine heart. Am. J. Physiol. Heart Circ. Physiol. 294, H2838–H2844 10.1152/ajpheart.00186.2008 18441205 PMC2863006

[56] Nithipatikom K., Moore J.M., Isbell M.A., Falck J.R. and Gross G.J. (2006) Epoxyeicosatrienoic acids in cardioprotection: ischemic versus reperfusion injury. Am. J. Physiol. Heart Circ. Physiol. 291, H537–H542 10.1152/ajpheart.00071.2006 16473964

[57] Seubert J., Yang B., Bradbury J.A., Graves J., Degraff L.M., Gabel S. (2004) Enhanced postischemic functional recovery in CYP2J2 transgenic hearts involves mitochondrial ATP-sensitive K+ channels and p42/p44 MAPK pathway. Circ. Res. 95, 506–514 10.1161/01.RES.0000139436.89654.c8 15256482

[58] Seubert J.M., Zeldin D.C., Nithipatikom K. and Gross G.J. (2007) Role of epoxyeicosatrienoic acids in protecting the myocardium following ischemia/reperfusion injury. Prostaglandins Other Lipid Mediat. 82, 50–59 10.1016/j.prostaglandins.2006.05.017 17164132 PMC2077836

[59] Guglielmino K., Jackson K., Harris T.R., Vu V., Dong H., Dutrow G. (2012) Pharmacological inhibition of soluble epoxide hydrolase provides cardioprotection in hyperglycemic rats. Am. J. Physiol. Heart Circ. Physiol. 303, H853–H862 10.1152/ajpheart.00154.2012 22865388 PMC3469704

[60] Wang H. and Eckel R.H. (2009) Lipoprotein lipase: from gene to obesity. Am. J. Physiol. Endocrinol. Metab. 297, E271–E288 10.1152/ajpendo.90920.2008 19318514

[61] Dijk W. and Kersten S. (2014) Regulation of lipoprotein lipase by Angptl4. Trends Endocrinol. Metab. 25, 146–155 10.1016/j.tem.2013.12.005 24397894

[62] Kersten S. (2014) Physiological regulation of lipoprotein lipase. Biochim. Biophys. Acta 1841, 919–933 10.1016/j.bbalip.2014.03.013 24721265

[63] Romeo S., Yin W., Kozlitina J., Pennacchio L.A., Boerwinkle E., Hobbs H.H. (2009) Rare loss-of-function mutations in ANGPTL family members contribute to plasma triglyceride levels in humans. J. Clin. Invest. 119, 70–79, 19075393 10.1172/JCI37118 PMC2613476

[64] Wang Y., McNutt M.C., Banfi S., Levin M.G., Holland W.L., Gusarova V. (2015) Hepatic ANGPTL3 regulates adipose tissue energy homeostasis. Proc. Natl. Acad. Sci. U.S.A. 112, 11630–11635 10.1073/pnas.1515374112 26305978 PMC4577179

[65] Tikka A. and Jauhiainen M. (2016) The role of ANGPTL3 in controlling lipoprotein metabolism. Endocrine 52, 187–193 10.1007/s12020-015-0838-9 26754661 PMC4824806

[66] Liu J., Afroza H., Rader D.J. and Jin W. (2010) Angiopoietin-like protein 3 inhibits lipoprotein lipase activity through enhancing its cleavage by proprotein convertases. J. Biol. Chem. 285, 27561–27570 10.1074/jbc.M110.144279 20581395 PMC2934623

[67] Shimamura M., Matsuda M., Yasumo H., Okazaki M., Fujimoto K., Kono K. (2007) Angiopoietin-like protein3 regulates plasma HDL cholesterol through suppression of endothelial lipase. Arterioscler. Thromb. Vasc. Biol. 27, 366–372 10.1161/01.ATV.0000252827.51626.89 17110602

[68] Minicocci I., Montali A., Robciuc M.R., Quagliarini F., Censi V., Labbadia G. (2012) Mutations in the ANGPTL3 gene and familial combined hypolipidemia: a clinical and biochemical characterization. J. Clin. Endocrinol. Metab. 97, E1266–E1275 10.1210/jc.2012-1298 22659251 PMC5393441

[69] Helgadottir A., Gretarsdottir S., Thorleifsson G., Hjartarson E., Sigurdsson A., Magnusdottir A. (2016) Variants with large effects on blood lipids and the role of cholesterol and triglycerides in coronary disease. Nat. Genet. 48, 634–639 10.1038/ng.3561 27135400 PMC9136713

[70] Musunuru K., Pirruccello J.P., Do R., Peloso G.M., Guiducci C., Sougnez C. (2010) Exome sequencing, ANGPTL3 mutations, and familial combined hypolipidemia. N. Engl. J. Med. 363, 2220–2227 10.1056/NEJMoa1002926 20942659 PMC3008575

[71] Stitziel N.O., Khera A.V., Wang X., Bierhals A.J., Vourakis A.C., Sperry A.E. (2017) ANGPTL3 deficiency and protection against coronary artery disease. J. Am. Coll. Cardiol. 69, 2054–2063 10.1016/j.jacc.2017.02.030 28385496 PMC5404817

[72] Ando Y., Shimizugawa T., Takeshita S., Ono M., Shimamura M., Koishi R. (2003) A decreased expression of angiopoietin-like 3 is protective against atherosclerosis in apoE-deficient mice. J. Lipid Res. 44, 1216–1223 10.1194/jlr.M300031-JLR200 12671033

[73] Dewey F.E., Gusarova V., Dunbar R.L., O’Dushlaine C., Schurmann C., Gottesman O. (2017) Genetic and pharmacologic inactivation of ANGPTL3 and cardiovascular disease. N. Engl. J. Med. 377, 211–221 10.1056/NEJMoa1612790 28538136 PMC5800308

[74] Graham M.J., Lee R.G., Brandt T.A., Tai L.J., Fu W., Peralta R. (2017) Cardiovascular and metabolic effects of ANGPTL3 antisense oligonucleotides. N. Engl. J. Med. 377, 222–232 10.1056/NEJMoa1701329 28538111

